# Reported Hearing Loss in Alzheimer’s Disease Is Associated With Loss of Brainstem and Cerebellar Volume

**DOI:** 10.3389/fnhum.2021.739754

**Published:** 2021-09-24

**Authors:** Daniel A. Llano, Susanna S. Kwok, Viswanath Devanarayan, The Alzheimer’s Disease Neuroimaging Initiative (ADNI)

**Affiliations:** ^1^Molecular and Integrative Physiology, University of Illinois at Urbana-Champaign, Urbana, IL, United States; ^2^Carle Neuroscience Institute, Urbana, IL, United States; ^3^Carle Illinois College of Medicine, Urbana, IL, United States; ^4^Beckman Institute for Advanced Science and Technology, Urbana, IL, United States; ^5^Eisai Inc., Woodcliff Lake, NJ, United States; ^6^Department of Mathematics, Statistics and Computer Science, University of Illinois at Chicago, Chicago, IL, United States

**Keywords:** presbycusis, cerebellum, auditory, brainstem, dementia, Alzheimer’s

## Abstract

Multiple epidemiological studies have revealed an association between presbycusis and Alzheimer’s Disease (AD). Unfortunately, the neurobiological underpinnings of this relationship are not clear. It is possible that the two disorders share a common, as yet unidentified, risk factor, or that hearing loss may independently accelerate AD pathology. Here, we examined the relationship between reported hearing loss and brain volumes in normal, mild cognitive impairment (MCI) and AD subjects using a publicly available database. We found that among subjects with AD, individuals that reported hearing loss had smaller brainstem and cerebellar volumes in both hemispheres than individuals without hearing loss. In addition, we found that these brain volumes diminish in size more rapidly among normal subjects with reported hearing loss and that there was a significant interaction between cognitive diagnosis and the relationship between reported hearing loss and these brain volumes. These data suggest that hearing loss is linked to brainstem and cerebellar pathology, but only in the context of the pathological state of AD. We hypothesize that the presence of AD-related pathology in both the brainstem and cerebellum creates vulnerabilities in these brain regions to auditory deafferentation-related atrophy. These data have implications for our understanding of the potential neural substrates for interactions between hearing loss and AD.

## Introduction

The increase in human lifespan afforded by modern medicine has revealed an emerging health crisis: the growing numbers of individuals with aging-related hearing loss (ARHL) and Alzheimer’s Disease (AD). These disorders strip away patients’ abilities to connect with their loved ones, either via verbal communication or by relating shared experiences. The number of individuals with these disorders is expected to grow because of the aging of our society. By 2050 it is estimated that approximately 1.5 billion people worldwide will be over the age of 65 (He et al., 2016), and approximately 40% of them will have ARHL (Nash et al., 2011) and 10% will have AD (Hebert et al., 2013).

A series of recent epidemiological studies has shown that elderly individuals with ARHL have a higher risk of developing aging-related cognitive decline, mild cognitive impairment (MCI) or AD (Lin et al., 2011a, b,c, 2013; Panza et al., 2015; Golub et al., 2017; Thomson et al., 2017; Ford et al., 2018). Across these studies, the increase in risk of developing cognitive impairment is approximately 50–100% above baseline, and this risk increase exists despite controlling for potentially confounding variables such as age, apolipoprotein E genotype and cardiovascular risk factors. In addition, hearing loss (HL) in AD is not only observed in the auditory periphery. In fact, patients with AD show evidence of more central, rather than peripheral, auditory dysfunction, though both are affected in this disorder (reviewed in Swords et al., 2018).

The finding that ARHL and AD are epidemiologically linked raises several questions with therapeutic implications. For example, it is not yet known if AD and ARHL are causally associated, or if a third, as yet unidentified factor, mediates this relationship. If they are causally linked, then efforts to mitigate one (e.g., aggressive early treatment of ARHL) may lower the incidence of the other. Currently less than 20% of individuals that could benefit from hearing aids use them (Popelka et al., 1998; Chien and Lin, 2012), suggesting that there is a large potential pool of individuals that could benefit from early intervention to limit the impact of ARHL, and therefore potentially lower the incidence of AD. Consistent with the potential benefits of early and aggressive intervention to treat ARHL is the finding that cochlear implantation may enhance overall cognitive function in elderly patients with hearing loss (Utoomprurkporn et al., 2020; Knopke et al., 2021; Mertens et al., 2021). The animal literature is generally supportive of the finding that induction of HL can cause more global cognitive dysfunction, but does not generally lead to a progressive degenerative phenotype (reviewed by Nadhimi and Llano, 2020). Alternatively, if ARHL and AD are linked by a third factor (e.g., brain inflammation, oxidative stress, metabolic dysfunction, etc.), then aggressive treatment of that third factor would be required to limit the impact of both disorders. Supporting this latter hypothesis is the recent finding of an interaction between ARHL, AD and serum phosphatidylcholine levels (Llano et al., 2020). Low blood phosphatidylcholine levels are an indicator of oxidative stress and inflammation (Jacobsson et al., 1990; Kurutas, 2015), and these levels were lowered in individuals with ARHL and AD, but not in controls (Llano et al., 2020), suggesting that the relationship between ARHL and AD may be mediated by factors common to both disorders. If a mediating factor is present, it is not yet clear how that factor impacts the brain or which brain regions may be most vulnerable.

Therefore, in the current study, we examined the anatomical substrates that may link AD and HL. Specifically, we examined volumetric magnetic resonance imaging (MRI) data from cognitively normal controls, MCI subjects and AD subjects with or without reports of HL using a publicly available dataset. We found several hindbrain regions (brainstem, bilateral cerebellum cortex and cerebellar white matter) that showed volume reductions in subjects with both reported HL and AD, but not in cognitively normal control or MCI subjects. This interaction between reported HL, AD diagnostic state and hindbrain volume loss suggests that the presence of AD pathology may reveal pathological interactions between the peripheral auditory system and structures in the early central auditory system. It is not yet known if an additional factor may explain these interactions.

## Materials and Methods

### Database

Data used in the preparation of this article were obtained from the Alzheimer’s Disease Neuroimaging Initiative (ADNI) database^1^. The ADNI was launched in 2003 as a public-private partnership, led by Principal Investigator Michael W. Weiner, MD. The primary goal of ADNI has been to test whether serial MRI, positron emission tomography (PET), other biological markers, and clinical and neuropsychological assessment can be combined to measure the progression of MCI and early AD. For up-to-date information, see www.adni-info.org. This study was registered under clinicaltrials.gov under ClinicalTrials.gov Identifier: NCT00106899. The study was conducted across multiple clinical sites and was approved by the Institutional Review Boards of all of the participating institutions. Informed written consent was obtained from all participants at each site. The following individual ethics boards approved the study: Albany Medical College Institutional Review Board, Boston University Medical Campus Institutional Review Board (BU IRB), Butler Hospital Institutional Review Board, Cleveland Clinic Institutional Review Board, Columbia University Institutional Review Board, Dartmouth-Hitchcock Medical Center Committee for the Protection of Human Subjects, Duke University Health System Institutional Review Board, Emory University Institutional Review Board Georgetown University Institutional Review Board, Human Investigation Committee Yale University School of Medicine, Human Subjects Committee, University of Kansas Medical Center, Indiana University Institutional Review Board, Research Compliance Administration, Institutional Review Board of Baylor College of Medicine, Institutional Review Board of the Mount Sinai School of Medicine, Johns Hopkins University School of Medicine Institutional Review Boards, Lifespan—Rhode Island Hospital Institutional Review Board, Mayo Clinic Institutional Review Board, Nathan Kline Institute Rockland Psychiatric Center Institutional Review Board (NKI RPC IRB), New York University Langone Medical Center School of Medicine, Institutional Review Board Human Research Program, Northwestern University Institutional Review Board Office, Office of the Washington University School of Medicine IRB (OWUMC IRB), Oregon Health and Science University Institutional Review Board, Partners Human Research Committee, Research Ethics Board Jewish General Hospital, Research Ethics Board Sunnybrook Health Sciences Centre, Roper St. Francis Institutional Review Board, Rush University Medical Center Institutional Review Board, Stanford University, Administrative Panel on Human Subjects in Medical Research, The Ohio State University Institutional Review Board, The University of Texas Southwestern Medical Center Institutional Review Board, UCLA Office of the Human Research Protection Program Institutional Review Board, UCSD Human Research Protections Program, University Hospitals Case Medical Center Institutional Review Board, University of Alabama at Birmingham Institutional Review Board, University of British Columbia, Clinical Research Ethics Board (CREB), University of California Davis Office of Research IRB Administration, University of California Irvine Office Of Research Institutional Review Board (IRB), University of California San Francisco Committee on Human Research (CHR), University of Iowa Institutional Review Board, University of Kentucky Office of Research Integrity, University of Michigan Medical School Institutional Review Board (IRBMED), University of Pennsylvania Institutional Review Board, University of Pittsburgh Institutional Review Board, University of Rochester Research Subjects Review Board (RSRB), University of South Florida Division of Research Integrity & Compliance, University of Southern California Health Science Campus Institutional Review Board, University of Western Ontario Research Ethics Board for Health Sciences Research Involving Human Subjects (HSREB), University of Wisconsin Health Sciences Institutional Review Board, Wake Forest University Institutional Review Board, Weill Cornell Medical College Institutional Review Board, Western Institutional Review Board and Western University Health Sciences Research Ethics Board. Data used for the analyses presented here were accessed on August 16, 2020.

### Clinical Diagnosis and Hearing Loss Assessment

AD was diagnosed using NINCDS/ADRDA criteria for probable AD (McKhann et al., 1984). MCI patients had a memory complaint, an abnormal score on the Logical Memory II subscale from the Wechsler Memory Scale, an MMSE score between 24 and 30 and a Clinical Dementia Rating scale score of 0.5. Cognitively normal subjects did not have a memory complaint, had a normal score on the Logical Memory II subscale and had a Clinical Dementia Rating scale score of zero. Hearing was not systematically measured in the ADNI database. Similar to previous reports (Xu et al., 2019; Llano et al., 2020), we used subjective hearing loss complaints found in the following datasheets: ADSXLIST.csv, BLSCHECK.csv, INITHEALTH.csv, MEDHIST.csv, NEUROEXM.csv, PHYSICAL.csv, RECBLLOG.csv, RECMHIST.csv. We used the search terms “hear,” “auditory,” “ear,” “deaf,” “presbycusis,” and “HOH (hard of hearing)” and eliminated those reports that were clearly not related to ARHL (e.g., skin cancer on ear, earwax, etc.), as well as eliminating entries that referred to tinnitus without mention of hearing loss as well as central processing disorder, and eliminated duplicates. These search terms are identical to those used by Xu et al. (2019) and Llano et al. (2020) and were selected prior to the data being seen. Subjects having a hearing complaint are labeled in this study as “reported hearing loss” or RHL. Other subjects are listed as “non-reported hearing loss” or NHL.

#### Imaging

MRI methods for ADNI have been reported previously in detail (Jack et al., 2008).^2^ In brief, scans at 1.5 or 3T were obtained across multiple sites and scanners (General Electric, Philips, Siemens). T1-weighted images were obtained using MP-RAGE (Magnetization Prepared Rapid Acquisition Gradient Echo) sequences and were obtained at multiple timepoints for individual subjects. Segmentation of brain regions was done using FreeSurfer software^3^ (Dale et al., 1999; Fischl et al., 1999).

### Statistical Methods

The effect of each of the 257 regional brain MRI features (volume/area/thickness) on RHL in AD, MCI and cognitively normal subjects was assessed via analysis of covariance (ANCOVA) after adjusting for gender and age as covariates. Subjects with absolute value of studentized residuals from this model exceeding 3 were identified as outliers and excluded from further analysis. The summary measures reported from this analysis include the fold change of the regional MRI feature for the subjects with RHL relative to the subjects with NHL, Cohen’s D statistic, covariates-adjusted significance (*p*-value), an estimate of the false discovery rate, q-value (Benjamini and Hochberg, 1995). MRI features with *q*-value < 0.1 were considered as statistically significant, which is aligned with published recommendations (Efron, 2007). In addition, we apply a criterion of an absolute value of Cohen’s D to be greater than 0.2 to ensure sufficient magnitudes of the statistically significant effects (Sawilowsky, 2009).

For the regional MRI features that are significantly different (*q* < 0.1) at baseline between RHL and NHL, the rate of change over time (slope) of the composite sum of these features were compared between the RHL and NHL subjects for each diagnostic group separately via a mixed effects model. In this model, Time (month) since first visit was included as a continuous covariate, RHL vs. NHL and the interaction of Time with RHL and NHL were included as fixed effects, and subjects were included as random effect. The quadratic trend of Time was also included and retained in the model if it was significant. The interaction effect from this model was used to assess whether the slope of the composite MRI feature over time was greater in RHL subjects relative to the NHL subjects, with the one-sided *p*-value < 0.05 used as the criteria for statistical significance. All analyses were carried out using R version 4.0.5 (R Development Core Team, 2014).

## Results

### Demographics

Data were obtained from a total of 725 subjects, of whom 229 were age-matched cognitively normal, 308 had MCI and 188 had AD. Among these subjects, 67 cognitively normal (29.2%), 92 MCI (29.8%) and 42 AD (22.3%) subjects reported HL. The proportions of subjects reporting HL was similar in all three groups (*p* > 0.05, Chi-Square). RHL subjects were on average older than NHL subjects among those that were diagnosed as MCI or AD (*p* < 0.05, Wilcoxon), but not significantly different among cognitively normal subjects. RHL subjects were more likely to be men than NHL subjects among those that were diagnosed as either MCI or AD (*p* < 0.05, Chi-Square), but not significantly different among the cognitively normal subjects. RHL and NHL subjects did not differ significantly with respect to Education level, apolipoprotein E4 (ApoE4) and Mini-mental status exam (MMSE) in all three diagnostic groups (cognitively normal, MCI and AD). See Table 1 for further details. Given the differences in age and sex in RHL vs. NHL subjects listed above, all subsequent analyses were adjusted for age and sex.

**TABLE 1:** Demographics.

		Cognitively normal (*n* = 229)		MCI (*n* = 308)		AD (*n* = 188)	
		NHL	RHL	NHL	RHL	NHL	RHL
	**N**	162	67	216	92	146	42
**Gender**	Female (n)	82	28	84	22*	77	13*
	Male (n)	80	39	132	70	69	29
**ApoE**	E4 (n)	38	23	122	43	98	27
	non-E4 (n)	124	44	94	49	48	15
**Age (years):** Mean (SD)		75.6 (4.9)	76.5 (5.2)	73.8 (7.5)	77.8* (6.1)	74.6 (7.7)	77.6* (5.9)
**Education (years):** Mean (SD)		15.8 (2.9)	16.6 (2.7)	15.6 (3)	15.8 (2.9)	14.8 (2.9)	14.5 (3.8)
**MMSE:** Mean (SD)		29.1 (1)	29.1 (1)	27 (1.7)	27 (1.8)	23.5 (1.9)	22.8 (2.4)

Values are mean (SD). RHL, reported hearing loss. NHL, no reported hearing loss. *p < 0.05 comparing RHL to NHL.

### Impact of Reported Hearing Loss in Cognitively Normal and Mild Cognitive Impairment Subjects

257 brain regions were examined and compared between cognitively normal subjects with or without RHL. After adjusting for age and sex, and controlling for false discovery rates, none of the brain regions demonstrated differences between RHL and NHL subjects. Similarly for MCI subjects, there were no differences in regional brain volumes between hearing RHL and NHL subjects (Tables 2, 3).

**TABLE 2:** Top 20 markers, based on *p* and *q*-values, differentiating RHL from NHL in cognitively normal subjects, corrected for age and gender.

Rank	Marker ID	Marker name	Ratio RHL/NHL	CohensD	p (Marker)	q (Marker)
1	ST60TA	Cortical Thickness Average of LeftTemporalPole	1.04	0.52	0.0007	0.1730
2	ST5SV	Volume (WM Parcellation) of CorpusCallosumMidPosterior	1.07	0.29	0.0053	0.6840
3	ST11SV	Volume (WM Parcellation) of LeftAccumbensArea	1.09	0.45	0.0178	0.7650
4	ST4SV	Volume (WM Parcellation) of CorpusCallosumMidAnterior	1.07	0.39	0.0202	0.7650
5	ST103TA	Cortical Thickness Average of RightParahippocampal	1.04	0.39	0.0209	0.7650
6	ST128SV	Volume (WM Parcellation) of WMHypoIntensities	0.92	–0.15	0.0414	0.7650
7	ST6SV	Volume (WM Parcellation) of CorpusCallosumPosterior	1.05	0.31	0.0427	0.7650
8	ST112SV	Volume (WM Parcellation) of RightPutamen	1.03	0.28	0.0467	0.7650
9	ST73CV	Volume (Cortical Parcellation) of RightCaudalAnteriorCingulate	1.03	0.14	0.0481	0.7650
10	ST59CV	Volume (Cortical Parcellation) of LeftSupramarginal	1.03	0.19	0.0483	0.7650
11	ST24CV	Volume (Cortical Parcellation) of LeftEntorhinal	1.05	0.25	0.0525	0.7650
12	ST7SV	Volume (WM Parcellation) of CSF	0.91	–0.45	0.0539	0.7650
13	ST113SA	Surface Area of RightRostralAnteriorCingulate	1.07	0.29	0.0567	0.7650
14	ST91CV	Volume (Cortical Parcellation) of RightInferiorTemporal	1.01	0.08	0.0649	0.7650
15	ST44CV	Volume (Cortical Parcellation) of LeftParahippocampal	1.07	0.49	0.0657	0.7650
16	ST43SA	Surface Area of LeftParacentral	1.02	0.2	0.0667	0.7650
17	ST12SV	Volume (WM Parcellation) of LeftAmygdala	1.03	0.23	0.0675	0.7650
18	ST99CV	Volume (Cortical Parcellation) of RightMiddleTemporal	1.04	0.24	0.0697	0.7650
19	ST72TA	Cortical Thickness Average of RightBankssts	1.01	0.15	0.0730	0.7650
20	ST102TA	Cortical Thickness Average of RightParacentral	1.02	0.17	0.0752	0.7650

*WM, white matter; CSF, cerebrospinal fluid.*

**TABLE 3:** Top 20 markers, based on *p* and *q*-values, differentiating HL from NHL in MCI subjects, corrected for age and gender.

Rank	Marker ID	Marker name	Ratio RHL/NHL	CohensD	p (Marker)	q (Marker)
1	ST106TA	Cortical Thickness Average of RightParsTriangularis	1.02	0.20	0.0019	0.4860
2	ST44SA	Surface Area of LeftParahippocampal	0.95	–0.38	0.0060	0.5720
3	ST45TA	Cortical Thickness Average of LeftParsOpercularis	1.01	0.07	0.0088	0.5720
4	ST36TA	Cortical Thickness Average of LeftLateralOrbitofrontal	1.00	0.05	0.0125	0.5720
5	ST121TA	Cortical Thickness Average of RightTransverseTemporal	1.05	0.30	0.0144	0.5720
6	ST72TA	Cortical Thickness Average of RightBankssts	1.01	0.15	0.0155	0.5720
7	ST106SA	Surface Area of RightParsTriangularis	0.95	–0.29	0.0156	0.5720
8	ST104TA	Cortical Thickness Average of RightParsOpercularis	1.01	0.09	0.0244	0.7510
9	ST102CV	Volume (Cortical Parcellation) of RightParacentral	1.02	0.12	0.0265	0.7510
10	ST68SV	Volume (WM Parcellation) of Non-WMHypoIntensities	0.85	–0.23	0.0382	0.7510
11	ST16SV	Volume (WM Parcellation) of LeftCaudate	0.97	–0.21	0.0389	0.7510
12	ST84CV	Volume (Cortical Parcellation) of RightFrontalPole	1.03	0.13	0.0431	0.7510
13	ST48SA	Surface Area of LeftPericalcarine	0.96	–0.22	0.0457	0.7510
14	ST25CV	Volume (Cortical Parcellation) of LeftFrontalPole	1.01	0.03	0.0501	0.7510
15	ST75SV	Volume (WM Parcellation) of RightCaudate	0.98	–0.13	0.0517	0.7510
16	ST110TA	Cortical Thickness Average of RightPrecentral	0.99	–0.09	0.0524	0.7510
17	ST23TA	Cortical Thickness Average of LeftCuneus	1.01	0.08	0.0536	0.7510
18	ST73TA	Cortical Thickness Average of RightCaudalAnteriorCingulate	1.01	0.10	0.0542	0.7510
19	ST54SA	Surface Area of LeftRostralAnteriorCingulate	0.95	–0.24	0.0565	0.7510
20	ST50SA	Surface Area of LeftPosteriorCingulate	0.99	–0.04	0.0595	0.7510

*WM, white matter.*

### Impact of Hearing Loss in Alzheimer’s Disease Subjects

**FIGURE 1:**
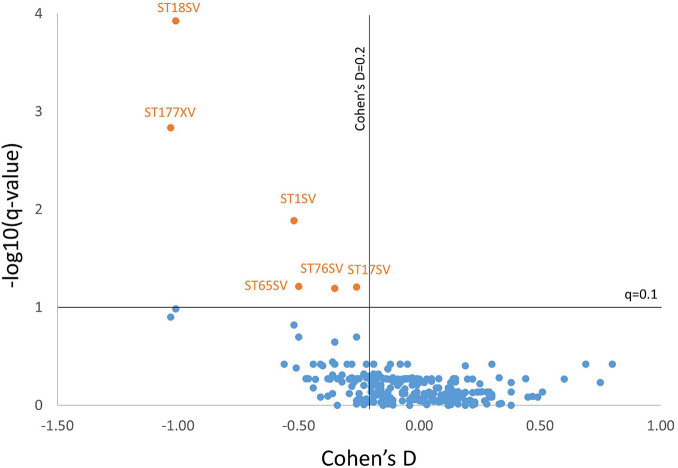
Volcano plot showing the Cohen’s D value vs. –log10(*q*-value) for all markers for AD subjects. Six markers met criteria for having a *q*-value of less than 0.1 (–log10(*q*-value) > 1) and having a Cohen’s D of above 0.2. Marker codes are shown. Full marker names can be found in Table 4.

**FIGURE 2:**
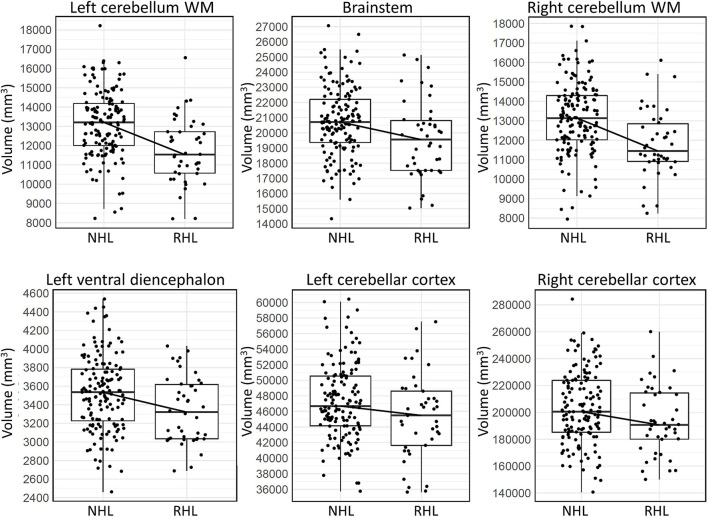
Box plots showing the median, first and third quartiles of the distributions, demonstrating differences in volumes of the six brain regions in AD subjects that significantly differentiated RHL from NHL subjects. WM, white matter.

After adjusting for age and sex, and after controlling for multiple comparisons, six brain regions were found to be significantly smaller in AD subjects with RHL compared to NHL subjects and had Cohen’s D values above 0.2. These brain regions were the volume of left and right cerebellar white matter, volume of the brain stem, volume of left ventral diencephalon, and the volume of left and right cerebellar cortex (see Figure 1 for volcano plot). Thus, five out of the six regions that differentiated reports of hearing loss from non-reported hearing loss were either in the cerebellum or brainstem, and all regions showed smaller volumes with an average effect size of −0.61 (0.33 SD) (see Table 4 and Figure 2 for boxplots).

**TABLE 4:** Top 20 markers, based on *p* and *q*-values, differentiating HL from NHL in AD subjects, corrected for age and gender.

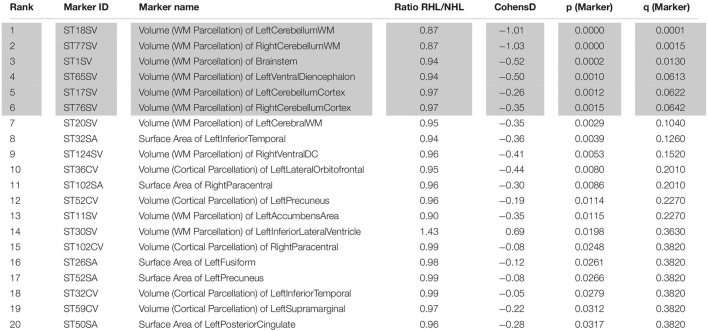

*Rows shaded in light gray met the q < 0.1 threshold. WM, white matte;, DC, diencephalon.*

For the six brain regions listed above that were significantly smaller in AD subjects with RHL, interaction of reported hearing status and disease diagnosis was assessed via analysis of covariance, after adjusting for age and gender. The interaction effect was significant in all of these brain regions, except for cerebellar cortex. However, *post hoc* evaluation of this interaction effect revealed that all these brain regions had significantly lower volume/area in only the AD subjects with RHL (*p* < 0.05), but not among the MCI and cognitively normal subjects. This relationship was not investigated further for ApoE4 carriers and non-carriers due to inadequate sample size of subgroups.

We then studied the rate of change over time of the six regional MRI features that were significantly different at baseline between RHL and NHL in AD patients. We took a composite sum of these features and compared their slopes over time between RHL and NHL in the cognitively normal, MCI and AD subjects separately. While this analysis revealed a greater rate of atrophy over time in the cognitively normal, MCI and AD subjects with RHL, this effect was only observed in the cognitively normal and did not reach significance in the MCI subjects (*p* = 0.029 and 0.084, respectively, Table 5). This finding implies that the greater atrophy seen in these brain regions at baseline in the AD subjects with RHL is a reflection of the faster atrophy over time in these brain regions in the cognitively normal subjects with RHL.

**TABLE 5:** Slopes of decline of composite volume score in NHL vs. RHL subjects, starting at cognitively normal, MCI or AD state.

Composite of six significant regional features between RHL and NHL in AD subjects at baseline	Slope of NHL (mm^3^/year)	Slope of HL (mm^3^/year)	Slope difference (RHL-NHL)	*p*-value for Slope of RHL > NHL
Cognitively normal	−86.57	−100.02	−13.44	0.029
MCI	−114.16	−124.58	−10.43	0.084
AD	−88.27	−98.13	−9.87	0.341

In addition, given the adverse cognitive impacts of psychotropic drug use in the elderly, particularly those with anticholinergic profiles, we examined whether use of anticholinergic drugs differed between RHL and NHL groups. Using updated Beers list criteria for anticholinergic drugs (Beers, 1997; Fick et al., 2003), we observed that 21/503 (4.01%) subjects with NHL and 9/201 (4.48%, *p* > 0.05) with RHL took anticholinergic drugs. These data suggest differential use of anticholinergic drugs is not likely to be responsible for differences in brain volumes observed in the current study.

## Discussion

In the current study, 257 brain regions were measured in 229 cognitively normal subjects, 308 MCI subjects and 188 AD subjects, and these subjects were further divided into those with self-report of hearing loss (RHL) or with no self-report of hearing loss (NHL). RHL was not associated with differences in brain volumes or surface areas in cognitively normal control or MCI subjects. In contrast, bilateral cerebellar volumes and brainstem volume were diminished in AD subjects with RHL compared to the NHL group. Two of these six brain regions (left and right cerebellar white matter) had effect sizes greater than 1.0, indicating a strong effect of reported hearing loss (Sawilowsky, 2009). A statistically significant interaction was found between these brain volumes, disease diagnosis and the presence of RHL. A composite score of all six brain regions also showed an average faster rate of decline over time in cognitively normal subjects with RHL compared to the NHL group. These data suggest that hearing impairment is associated with accelerated volume loss in the brainstem and cerebellum in AD subjects, which are both regions that receive substantial auditory input. These findings are discussed below.

### Limitations in the Study

This study employed subjective reports of HL, rather than objectively measured HL, such as can be measured with a pure-tone audiogram or auditory brainstem response. Use of subjective measures was required because hearing was not objectively measured in this dataset. Previous work has shown that subjective hearing loss shows an approximately 65–77% concordance rate with objectively measured hearing loss (Kamil et al., 2015). Thus, it is possible that subjects in the NHL group, in fact had some degree of loss of hearing, or vice-versa. The likely net impact of using subjective, rather than objective, metrics of hearing loss is to dilute the impact of loss of hearing on the differences between the measured brain volumes and surface areas in the two groups. Despite this necessary uncertainty in group assignment, previous studies of the ADNI dataset have used the same approach and revealed RHL-related differences across a range of biomarkers (Xu et al., 2019; Llano et al., 2020). These data suggest the impact of HL is robust to the diluting effect of assignment ambiguity, but that future biomarker studies in dementing illness should consider incorporation of objective measures of hearing impairment into their array of clinical tests.

### Implications of This Study

Similar to previous work (Xu et al., 2019), when subjects are stratified by disease state, no relationship was observed between reports of HL and hippocampal volume, which has traditionally been inversely associated with AD disease state (Gosche et al., 2002). With the majority of literature focusing on shared temporal lobe dysfunction, AD and ARHL together have not been described in the context of shared brainstem and cerebellar neuropathology. However, cerebellar and brainstem changes related to Aβ-pathology have been noted for over 100 years. As early as 1911 Bielschowsky observed, and was subsequently verified by Braak et al. in 1989, that amyloid plaques and tau tangles were found in the cerebellum (Bielschowsky, 1911; Braak et al., 1989). Recent MRI studies have shown progressive cerebellar gray matter reduction throughout the course of AD whereby involvement of the vermis and paravermian areas of the anterior (I–V) and posterior (VI) cerebellar lobes was noted in early AD and additional atrophy in the posterior hemispheric lobe (VI) and crus (I) in later stages (Guo et al., 2016; Gellersen et al., 2017; Jacobs et al., 2018; Toniolo et al., 2018, reviewed in Devita et al., 2021). Likewise, early studies suggested brainstem involvement in AD, and have also noted that brainstem pathology may precede pathology in other regions more typically associated with AD, such as hippocampus and neocortex (Rüb et al., 2001; Simic et al., 2009; Grinberg et al., 2011; Ehrenberg et al., 2017).

The presence of AD-related pathology in the brainstem and cerebellum suggests that these brain regions may be vulnerable in the setting of an additional insult, such as peripheral deafferentation. Both brain regions receive substantial input from the peripheral auditory system. For example, the eighth cranial nerve provides dense input to the cochlear nucleus which is situated at the pontomedullary junction. The cochlear nucleus then sends projections throughout the brainstem to the superior olive and nuclei of the lateral lemniscus (Santi and Mancini, 1998). The cerebellum also receives dense acoustic input. Anatomic connections between the cerebellum and auditory pathways have been documented in multiple species. For example, afferent anatomic connections from the cochlear nuclei and inferior colliculus have been described to extend to the pons, inferior olivary nucleus, vermis, and the dentate nuclei (Snider and Stowell, 1944; Aitkin and Boyd, 1975; Huang et al., 1982; Wang et al., 1991; McLachlan and Wilson, 2017). Additionally, projections from the inferior colliculus synapse onto the pontine nuclei and travel through the middle cerebral peduncle to connect to the cerebellar cortex (Aitkin and Boyd, 1978; Huffman and Henson, 1990; McLachlan and Wilson, 2017). These anatomical connections between the auditory system and cerebellum likely represent the substrate underlying cerebellar activation seen during auditory tasks in functional imaging studies (Ackermann, 2008; Petacchi et al., 2011; Schwartze and Kotz, 2016).

Thus, we speculate that loss of afferent acoustic input, in the setting of pre-existing AD pathology, induces downstream atrophy of synaptic targets in the brainstem and cerebellum. This atrophy is likely not seen in the cognitive-normal and MCI subjects because of the lower levels of AD pathology present in these populations. It is notable that brain regions associated with higher acoustic processing, such as regions of the superior temporal gyrus, which houses the auditory cortex, did not show this relationship, as has been seen in a previous study (Ren et al., 2018). Note that Ren et al. (2018) did not examine changes in the auditory brainstem or cerebellum. We note that another previous study found that temporal cortical atrophy was primarily driven by aging, rather that hearing loss (Profant et al., 2014). Given that temporal cortical regions receive less direct synaptic input from the auditory periphery than brainstem and certain regions of the cerebellum, such as the paraflocculus, they may be less prone to show deafferentation-related changes. It is also possible that floor effects may be present in our AD population such that AD patients may have atrophy of these brain regions beyond a degree which may be differentiated based on HL.

## Conclusion

Thus, in the current study, we observed that in the context of AD, RHL was associated with lowered volumes of the brainstem and cerebellum, as well as faster rates of declines in these regions. These data suggest that pathological changes that occur in the brainstem and cerebellum in AD increase the vulnerability of these regions to auditory deafferentation-related pathology. Given the growing literature suggesting that ARHL may predispose to AD, it is possible that acoustic deafferentation may trigger brainstem pathology. Given early pathological changes that occur in the brainstem in AD (Rüb et al., 2001; Simic et al., 2009), it is possible that HL may lead to pathological reorganization at the level of the brainstem and cerebellum, which, in vulnerable patients, may lead to broader AD pathology. Overall, our understanding of the cerebellum as it relates to either hearing or AD remains limited, and the analysis of both pathologies as they relate to changes in cerebellar volume was described for the first time by this study. In addition, the number of subjects in the AD with RHL group was relatively small (*n* = 42), and too small to parse effects of other important factors such as ApoE4 status. Thus, future work, particularly large-scale studies where both hearing levels and MRI volumes are quantitatively measured, will be needed to examine the relationship between hearing impairment and brain volume changes more closely.

## Data Availability Statement

Publicly available datasets were analyzed in this study. This data can be found here: http://adni.loni.usc.edu/.

## Ethics Statement

The study was conducted across multiple clinical sites and was approved by the Institutional Review Boards of all of the participating institutions. Informed written consent was obtained from all participants at each site. The following individual ethics boards approved the study: Albany Medical College Institutional Review Board, Boston University Medical Campus Institutional Review Board (BU IRB), Butler Hospital Institutional Review Board, Cleveland Clinic Institutional Review Board, Columbia University Institutional Review Board, Dartmouth-Hitchcock Medical Center Committee for the Protection of Human Subjects, Duke University Health System Institutional Review Board, Emory University Institutional Review Board Georgetown University Institutional Review Board, Human Investigation Committee Yale University School of Medicine, Human Subjects Committee, University of Kansas Medical Center, Indiana University Institutional Review Board, Research Compliance Administration, Institutional Review Board of Baylor College of Medicine, Institutional Review Board of the Mount Sinai School of Medicine, Johns Hopkins University School of Medicine Institutional Review Boards, Lifespan—Rhode Island Hospital Institutional Review Board, Mayo Clinic Institutional Review Board, Nathan Kline Institute Rockland Psychiatric Center Institutional Review Board (NKI RPC IRB), New York University Langone Medical Center School of Medicine, Institutional Review Board Human Research Program, Northwestern University Institutional Review Board Office, Office of the Washington University School of Medicine IRB (OWUMC IRB), Oregon Health and Science University Institutional Review Board, Partners Human Research Committee, Research Ethics Board Jewish General Hospital, Research Ethics Board Sunnybrook Health Sciences Centre, Roper St. Francis Institutional Review Board, Rush University Medical Center Institutional Review Board, Stanford University, Administrative Panel on Human Subjects in Medical Research, The Ohio State University Institutional Review Board, The University of Texas Southwestern Medical Center Institutional Review Board, UCLA Office of the Human Research Protection Program Institutional Review Board, UCSD Human Research Protections Program, University Hospitals Case Medical Center Institutional Review Board, University of Alabama at Birmingham Institutional Review Board, University of British Columbia, Clinical Research Ethics Board (CREB), University of California Davis Office of Research IRB Administration, University of California Irvine Office Of Research Institutional Review Board (IRB), University of California San Francisco Committee on Human Research (CHR), University of Iowa Institutional Review Board, University of Kentucky Office of Research Integrity, University of Michigan Medical School Institutional Review Board (IRBMED), University of Pennsylvania Institutional Review Board, University of Pittsburgh Institutional Review Board, University of Rochester Research Subjects Review Board (RSRB), University of South Florida Division of Research Integrity & Compliance, University of Southern California Health Science Campus Institutional Review Board, University of Western Ontario Research Ethics Board for Health Sciences Research Involving Human Subjects (HSREB), University of Wisconsin Health Sciences Institutional Review Board, Wake Forest University Institutional Review Board, Weill Cornell Medical College Institutional Review Board, Western Institutional Review Board and Western University Health Sciences Research Ethics Board. The patients/participants provided their written informed consent to participate in this study.

## Author Contributions

DL and VD conceived of the project. DL, SK, and VD wrote the manuscript. VD did data analysis. All authors contributed to the article and approved the submitted version.

## Conflict of Interest

VD was an employee of Eisai Pharmaceuticals. The remaining authors declare that the research was conducted in the absence of any commercial or financial relationships that could be construed as a potential conflict of interest.

## Publisher’s Note

All claims expressed in this article are solely those of the authors and do not necessarily represent those of their affiliated organizations, or those of the publisher, the editors and the reviewers. Any product that may be evaluated in this article, or claim that may be made by its manufacturer, is not guaranteed or endorsed by the publisher.

## Footnotes

1
adni.loni.usc.edu

2
http://adni.loni.usc.edu/methods/documents/mri-protocols/

3
https://surfer.nmr.mgh.harvard.edu/
